# Turnover Intention and Associated Factors Among Night‐Shift Midwives in China: A Cross‐Sectional Study

**DOI:** 10.1155/jonm/5081800

**Published:** 2026-05-15

**Authors:** Dongning He, Jinling Zhang, Jingbo He, Jinyu Zuo, Yangxue Meng, Na Li, Yan Huang, Mingxin Zhu, Guoyu Wang, Biru Luo

**Affiliations:** ^1^ Department of Nursing, West China Second University Hospital, Sichuan University/West China School of Nursing, Sichuan University, No. 20 Section 3 People’s South Road Wuhou District, Chengdu City, Sichuan Province, China, scu.edu.cn; ^2^ Key Laboratory of Birth Defects and Related Disease of Women and Children (Sichuan University), Ministry of Education, No. 20 Section 3 People’s South Road Wuhou District, Chengdu City, Sichuan Province, China, meb.gov.tr; ^3^ Department of Obstetric Nursing, West China Second University Hospital, Sichuan University/West China School of Nursing, Sichuan University, No. 20 Section 3 People’s South Road Wuhou District, Chengdu City, Sichuan Province, China, scu.edu.cn; ^4^ West China School of Nursing, Sichuan University, No. 24 South Section First Ring Road Wuhou District, Chengdu City, Sichuan Province, China, scu.edu.cn

**Keywords:** midwifery, personnel management, shift work schedule, turnover intention

## Abstract

**Objective:**

Midwives play a crucial role in maternal and neonatal health. However, there is a growing global shortage of midwives, and the high turnover rate is a key contributing factor. The research focusing on the turnover intention of night‐shift midwives is limited in China.

**Methods:**

This cross‐sectional study was conducted from January to December 2023 across 493 public hospitals in mainland China, with 1588 night‐shift midwives participating. A self‐designed questionnaire collected sociodemographic information and night‐shift work‐related characteristics. Turnover intention was measured using the Turnover Intention Scale, and sleep quality was assessed using the Pittsburgh Sleep Quality Index. Multiple linear regression analysis was conducted to identify factors influencing turnover intention.

**Results:**

A proportion of 49.7% (*n* = 789) of night‐shift midwives reported turnover intentions. Lower turnover intention was associated with being married, having better health status, good sleep quality, and ≥ 15 years of work experience. Higher satisfaction with shift scheduling and overall work were also associated with lower turnover intention. Conversely, completing additional work during rest time after night shifts and experiencing job burnout were associated with higher turnover intentions.

**Conclusions:**

High turnover intention among night‐shift midwives represents a significant challenge for maternal health services in China. Hospital administrators may consider prioritizing shift scheduling systems, regularly monitoring job burnout and satisfaction levels, and implementing targeted interventions to reduce turnover and ensure workforce sustainability.

## 1. Introduction

Midwives play a vital role in safeguarding maternal and neonatal health. Evidence from 461 systematic reviews indicates that midwife‐led care can improve 56 maternal and neonatal health outcomes [1] and is estimated to prevent up to 4.3 million deaths annually [2]. Midwives provide care during childbirth and throughout the pre‐pregnancy, pregnancy, and postpartum periods [3]. In addition, their contributions extend to health counseling, education, and women’s empowerment initiatives [4]. However, the global midwifery workforce faces a rising turnover rate [5, 6] and a severe shortage of personnel [2]. The World Health Organization projects a shortage of 310,000 midwives worldwide by 2030 [7, 8]. Studies indicate that this shortage may adversely affect maternal and neonatal care during the perinatal period [9]. Expanding the midwifery workforce has become a critical priority for global healthcare systems [10].

Research indicates that high turnover rates significantly contribute to the midwife shortage [5]. Consequently, researchers have focused on key factors influencing turnover, with rotational work being critical [11, 12]. Despite variations in rotational work schedules across countries, night shifts remain unavoidable for midwives [13]. Studies show that long‐term night shift work may negatively affect midwives’ physical and mental health [14, 15]. It has been reported that night shift work reduces sleep quality [16, 17], leading to fatigue [18], anxiety, and depression [19], and increases the risk of metabolic diseases such as obesity and diabetes [20], as well as cardiovascular disease [21]. Additionally, long‐term night shifts may impair midwives’ concentration and reaction times [22], thereby adversely affecting work performance and occupational safety [23, 24]. As fatigue and job burnout accumulate, midwives may develop negative attitudes toward their work, which in turn increases turnover intention [25, 26].

Turnover intention refers to an individual’s attitude toward their current profession, indicating their likelihood of staying in or leaving the profession. Although turnover intention differs from actual turnover, research shows that high turnover intention often signals an increased risk of workforce attrition, serving as a key indicator of workforce stability [27–29]. Numerous countries have reported midwives’ turnover intention. Studies have primarily focused on Europe, North America, Australia, and other developed nations, with some African developing countries also reporting similar trends. Research indicates that 33.3%–53% of midwives in developed countries exhibit turnover intention [30–32], compared to 39%–87.2% in African countries [33–35]. The substantial variation in turnover intention rates between developed and African countries highlights the complexity of the issue. This complexity stems from variations in midwives’ practice methods, work locations, and environments across countries [30, 31, 36, 37]. These contextual differences influence midwives’ turnover intention. Therefore, some reasons for turnover intention may not be suitable for different countries [38].

Amid the global midwife shortage and growing attention to turnover intention, Chinese midwives face significant challenges. Over the past decade, China’s birth rate has fluctuated due to the two‐child and three‐child policies, with a recent declining trend [39]. However, the latest national data indicate that China has only 3.3 nurses (including midwives) per 1000 people [40]. This proportion remains below the global average [41], indicating a persistent shortage of midwives in China. In China’s healthcare system, midwives are specialized nurses working primarily in hospital delivery rooms, which partly explains their inclusion in national nursing workforce statistics. Unlike many Western countries where midwifery constitutes an independent profession with separate regulatory frameworks, Chinese midwives operate within the nursing professional structure while assuming specialized responsibilities for maternal and neonatal care [42]. According to national healthcare regulations, midwives are authorized to provide obstetric care, including labor monitoring, normal delivery assistance, and immediate newborn care within their defined scope of practice [43]. To ensure continuous maternal care coverage, many midwives are required to undertake night shifts as part of their rotational duties. This integrated professional model creates unique occupational characteristics and demands that may influence turnover intention among this specialized workforce. However, research on midwives’ turnover rate or intention is scarce, with even less focus on night‐shift midwives and their influencing factors. Therefore, we conducted a national cross‐sectional survey among night‐shift midwives in China. This study aimed to assess the level of turnover intention and examine its associated factors, including sleep quality and work‐related factors. The findings may provide evidence for clinical managers and policymakers to improve working environments and develop targeted retention strategies.

## 2. Methods

### 2.1. Setting

This cross‐sectional study, conducted from January to December 2023 in public hospitals across mainland China, aimed to investigate turnover intention among night‐shift midwives and the associated factors. In this study, hospital levels were classified according to the Chinese Hospital Classification Management Standards. Hospitals were categorized into two types: general hospitals, providing comprehensive health services (e.g., internal medicine, surgery, obstetrics, and pediatrics), and specialized hospitals, focusing on women’s and children’s health services. The night shift modes for midwives were mainly divided into four types. Mode 1: A complete night shift cycle consists of an early night shift (17:00–04:00) and a late night shift (22:00–08:00). Mode 2: A full‐night shift (18:00–08:00). Mode 3: A complete night shift cycle includes an early night shift, a late night shift, and a full‐night shift. Mode 4: Continuous work for at least 24 h. The time ranges in parentheses indicate possible scheduling windows within which hospitals arrange shifts. They represent the earliest start and latest finish times for each shift type. Actual working hours vary as shown in Table 1. For Mode 3, the total duration represents the cumulative hours across one complete cycle of all three shift types. Midwives do not work night shifts exclusively but are required to work day shifts (typically 8:00–20:00, lasting 8–12 h) according to the rotation schedule before each night shift. Table 1 provides detailed information on average working hours, post‐shift rest time, and intervals between shifts for each night shift mode.

**TABLE 1:** Characteristics of different night‐shift modes of midwives.

	Mode 1	Mode 2	Mode 3	Mode 4
Early night shift duration, h	7.40 ± 1.20	—	7.66 ± 1.32	—
Late night shift duration, h	8.02 ± 1.39	—	8.10 ± 1.25	—
Full‐night shift duration, h	—	12.40 ± 2.69	13.79 ± 3.18	—
Total night shift duration, h	15.69 ± 1.46	12.40 ± 2.69	29.63 ± 3.35	25.03 ± 1.09
Rest time after each night shift, day	2.04 ± 0.62	1.84 ± 0.54	2.78 ± 0.58	2.84 ± 0.56
Interval between two night shifts, day	4.71 ± 0.89	3.99 ± 0.57	6.78 ± 0.56	5.55 ± 0.75

### 2.2. Participants

This study used convenience sampling to recruit participants from public hospitals served by the 10 standardized midwifery training bases of the China Maternal and Child Health Association. These bases are evenly distributed across the eastern, central, and western regions of mainland China. The inclusion criteria were: (1) registered midwives working in delivery rooms; (2) able to independently complete night shift duties without continuous supervision or guidance from clinical mentors; (3) having at least 6 months of night shift work experience; (4) possessing normal listening, reading, and writing skills; and (5) volunteering to participate and providing informed consent. The exclusion criteria were: (1) currently in the process of resigning and (2) diagnosed with psychiatric disorders (e.g., depression, anxiety) that impair work performance or study participation.

### 2.3. Measurement

#### 2.3.1. Sociodemographic and Work‐Related Characteristics

We collected the sociodemographic characteristics through a self‐designed questionnaire, including gender, age, health status, years of experience as a midwife, and other relevant factors. In addition, we gathered information on night‐shift‐related characteristics. These included night shift mode (Mode 1–4), other tasks during night shifts (e.g., surgical assistance, neonatal care), the ability to take short rests during night shifts, whether shifts could be completed on time, and conditions during post‐shift rest periods (e.g., being on‐call or required to complete other work). We also assessed satisfaction with rotation schedules, self‐reported job burnout (single‐item), and overall job satisfaction.

#### 2.3.2. Turnover Intention Scale

This study used the Turnover Intention Scale, developed by Mobley [44] and revised by Hom et al. [45]. The scale comprises 4 items and uses a 5‐point Likert scale, ranging from 1 (strongly disagree) to 5 (strongly agree). The total score ranges from 4 to 20, with higher scores reflecting stronger turnover intention [46]. This scale does not have a clearly defined cutoff score. We used the sample mean as a cutoff to report the proportion of midwives with turnover intention, based on a previous study: participants scoring ≥ the sample mean were classified as having turnover intention [34]. This cutoff is not a validated threshold and was used solely for descriptive analysis. This scale has been applied in nursing populations and has shown acceptable reliability and validity in prior studies [47, 48]. Cronbach’s alpha for this scale in our study was 0.743.

#### 2.3.3. Pittsburgh Sleep Quality Index

The Pittsburgh Sleep Quality Index was used to assess sleep quality of midwives. It was developed by Buysse et al. as a self‐assessment tool to evaluate various aspects of sleep [49]. It consists of 19 items grouped into 7 dimensions, each scored from 0 to 3. The total score ranges from 0 to 21, with higher scores indicating poorer sleep quality. A cutoff score of 7 was used in this study to classify sleep quality as either good or poor [50]. The Pittsburgh Sleep Quality Index has been widely used among healthcare workers, and prior studies in nurses and midwives have reported acceptable reliability and validity [50, 51]. This scale’s Cronbach’s alpha was 0.765.

### 2.4. Data Collection

We used Wenjuan Xing, an online survey platform widely used in China, to create an online survey. First, researchers emailed the study objectives and questionnaire content to the managers of standardized midwifery training bases. After reviewing and approving the materials, the managers contacted the obstetric ward managers of hospitals associated with the bases via email or WeChat (a social application in China). They then collected the email addresses of those willing to participate. The main researchers provided training to the three investigators, who were responsible for communicating with the midwifery training bases in the eastern, central, and western regions, respectively. The investigators were trained to clearly explain the questionnaire’s filling requirements to ensure that the managers and midwives understood them. The investigators then sent the questionnaire link and inclusion and exclusion criteria to the obstetric ward managers. The managers were asked to distribute the questionnaire to eligible midwives. If the managers or midwives had any questions about the questionnaire, they could contact the investigators via email, telephone, or WeChat. Before filling out the questionnaires, participants viewed a page explaining the study objectives and provided informed consent via digital signature. Participants could decide whether to join and withdraw at any time. As the survey link was disseminated through hospital managers, we were unable to determine the exact number of eligible midwives approached or calculate the response rate. We received 1759 questionnaires across 493 hospitals of various levels and types. Two independent investigators reviewed the completed questionnaires to check for logical inconsistencies or missing answers, and questionnaires with more than 20% missing data were excluded (*n* = 171). Finally, 1588 valid questionnaires remained for analysis, with a valid questionnaire proportion of 90.3%. The sample size was justified based on a commonly used rule of thumb for multiple linear regression (at least 10–15 participants per predictor). With 20 candidate predictors, the minimum required sample size was estimated as 20 × 15 = 300. The final analytic sample (*n* = 1588) exceeded this requirement.

### 2.5. Data Statistics

Data were analyzed using IBM SPSS version 27.0. In this study, the time data for different night shift modes were continuous variables that followed normal distributions and were presented as the mean ± standard deviation (SD). Sociodemographic and night‐shift work‐related characteristics were categorical variables, shown as frequencies (*n*) and percentages (%). The total scores of turnover intention and sleep quality scales followed normal distributions, but individual items of the turnover intention scale did not. Thus, scale scores were presented using the mean ± SD, as well as medians and interquartile ranges (IQR). Non‐parametric tests were used to examine associations between categorical variables and turnover intention. The Mann–Whitney *U* test was used for two‐group comparisons, and the Kruskal–Wallis H test was applied for comparisons among multiple groups. Multiple linear regression was conducted to identify factors influencing turnover intention, with variables showing *p* < 0.05 in univariate analyses entered into the model. For multi‐category variables, groups with the lowest turnover intention score were chosen as a reference when possible. All statistical analyses were two‐tailed, and *p* < 0.05 was considered statistically significant.

### 2.6. Ethics Approval

This study was conducted in accordance with the Declaration of Helsinki and was approved by the Ethics Committee of West China Second University Hospital, Sichuan University. The ethics approval number was 2022 (322). All participants provided informed consent.

## 3. Results

### 3.1. The Sociodemographic Characteristics of Participants

A total of 1588 participants were finally included in the study. Most participants were female (99.6%). A total of 235 participants reported excellent health, while 77 reported poor health status. The total score of the Pittsburgh Sleep Quality Index was 9.40 ± 3.79. According to the cutoff, 1071 (67.4%) of midwives had poor sleep quality. The majority of participants held undergraduate degrees (81.4%); 69% of participants were from public tertiary A hospitals, and 64.6% (*n* = 1026) were from general hospitals. The sociodemographic characteristics are shown in Table 2, and significant differences in turnover intention are summarized in Section 3.3.

**TABLE 2:** Sociodemographic characteristics and turnover intention of midwives (*N* = 1588).

Variables	*N* (%)	Turnover intention mean ± SD	*Z*/*H*	*p* value
Gender			−0.247^a^	0.805
Male	6 (0.4)	12.00 ± 3.80		
Female	1582 (99.6)	11.44 ± 2.69		
Age, yr			6.352^b^	0.096
< 25	66 (4.2)	11.82 ± 2.63		
25∼	1036 (65.2)	11.53 ± 2.68		
35∼	446 (28.1)	11.28 ± 2.76		
≥ 45	40 (2.5)	10.68 ± 2.53		
Marital status			−4.044^a^	< 0.001
Single	353 (22.2)	11.94 ± 2.69		
Married	1235 (77.8)	11.30 ± 2.68		
Fertility status			−2.625^a^	0.009
No child	1075 (67.7)	11.32 ± 2.69		
One child and above	513 (32.3)	11.72 ± 2.70		
Health status			121.193^b^	< 0.001
Excellent	235 (14.8)	10.67 ± 2.77		
Good	561 (35.3)	10.85 ± 2.58		
Fair	715 (45.0)	11.94 ± 2.56		
Poor	77 (4.9)	13.55 ± 2.54		
Sleep quality			−10.519^a^	< 0.001
Poor (> 7)	1071 (67.4)	11.93 ± 2.61		
Good (≤ 7)	517 (32.6)	10.44 ± 2.60		
Educational level			2.142^b^	0.343
Junior college and below	276 (17.4)	11.29 ± 2.76		
Undergraduate	1293 (81.4)	11.47 ± 2.69		
Master and above	19 (1.2)	11.84 ± 2.14		
Hospital level			14.429^b^	< 0.001
Public tertiary A hospital	1096 (69.0)	11.31 ± 2.65		
Public tertiary B hospital	210 (13.2)	11.87 ± 2.91		
Public secondary hospital and below	282 (17.8)	11.66 ± 2.67		
Hospital type			−0.665^a^	0.506
General hospital	1026 (64.6)	11.41 ± 2.69		
Specialized hospital	562 (35.4)	11.51 ± 2.72		
Years of experience as a midwife, yr			21.695^b^	< 0.001
< 5	381 (24.0)	11.45 ± 2.72		
5∼	593 (37.3)	11.46 ± 2.70		
10∼	393 (24.8)	11.78 ± 2.56		
≥ 15	221 (13.9)	10.80 ± 2.79		
Professional title			7.719^b^	0.052
Junior midwife	173 (10.9)	11.53 ± 2.85		
Senior midwife	747 (47.0)	11.51 ± 2.68		
Supervising midwife	621 (39.1)	11.42 ± 2.68		
Associate chief midwife and above	47 (3.0)	10.43 ± 2.58		

^a^ *Z*, Mann–Whitney *U* test.

^b^ *H*, Kruskal–Wallis H test.

### 3.2. Night‐Shift Work‐Related Characteristics of Participants

The data showed that more than half of the participants worked in Mode 2, and only 5.6% worked in Mode 4. Additionally, 49.9% were satisfied with the current rotation schedule, 79.8% experienced burnout, and 40.1% were satisfied with their overall work. All night‐shift work‐related characteristics are presented in Table 3. As shown in Table 3, turnover intention varied by night‐shift work‐related characteristics.

**TABLE 3:** Night‐shift work‐related characteristics and turnover intention of midwives (*N* = 1588).

Variables	*N* (%)	Turnover intention mean ± SD	*Z*/*H*	*p* value
Night shift mode			26.544^b^	< 0.001
Mode 1	431 (27.2)	11.00 ± 2.63		
Mode 2	890 (56.0)	11.48 ± 2.62		
Mode 3	178 (11.2)	12.09 ± 3.04		
Mode 4	89 (5.6)	11.93 ± 2.75		
Other tasks during the night shift			−5.511^a^	< 0.001
No	555 (34.9)	10.94 ± 2.55		
Yes	1033 (65.1)	11.72 ± 2.73		
Ability to take short rest breaks during night shift			−0.595^a^	0.552
No	1197 (75.4)	11.42 ± 2.71		
Yes	391 (24.6)	11.52 ± 2.65		
Finish night shift on time			−6.542^a^	< 0.001
No	794 (50.0)	11.87 ± 2.71		
Yes	794 (50.0)	11.03 ± 2.62		
On‐call during post‐shift rest			−6.784^a^	< 0.001
No	743 (46.8)	10.96 ± 2.56		
Yes	845 (53.2)	11.88 ± 2.74		
Additional work during post‐shift rest			−8.543^a^	< 0.001
No	880 (55.4)	10.97 ± 2.51		
Yes	708 (44.6)	12.04 ± 2.80		
Satisfied with rotation schedule			−15.803^a^	< 0.001
No	796 (50.1)	12.47 ± 2.49		
Yes	792 (49.9)	10.42 ± 2.50		
Job burnout			−8.255^a^	< 0.001
No	321 (20.2)	10.37 ± 2.28		
Yes	1267 (79.8)	11.72 ± 2.73		
Overall job satisfaction			−4.333^a^	< 0.001
No	952 (59.9)	11.69 ± 2.73		
Yes	636 (40.1)	11.08 ± 2.61		

Abbreviation: SD, Standard deviation.

^a^ *Z*, Mann–Whitney *U* test.

^b^ *H*, Kruskal–Wallis H test.

### 3.3. Turnover Intention of Participants With Different Characteristics

The participants’ total turnover intention score was 11.45 ± 2.70, with a median of 11 (Table 4). Using the sample mean (11.45) as the cutoff value, 789 (49.7%) night‐shift midwives were classified as having turnover intention. Comparisons of turnover intention across sociodemographic and night‐shift work‐related characteristics are presented in Tables 2 and 3, respectively. The results showed statistically significant differences in turnover intention based on marital status, fertility status, health status, sleep quality, hospital levels, and years of experience as a midwife (all *p* < 0.05). Significant differences were also observed across night‐shift work‐related characteristics. There were significant differences in turnover intention across night shift modes, with Mode 1 showing the lowest scores and Mode 3 the highest. Except for the ability to take short rest breaks during night shifts, turnover intention scores were significantly higher among participants who had additional tasks during night shifts or could not finish their work on time. Higher scores were also observed among those who were required to be on‐call, needed to complete other work during post‐shift rest periods, reported job burnout, or were dissatisfied with the current rotation schedule or overall work (all *p* < 0.001).

**TABLE 4:** Turnover intention scale score of midwives (*N* = 1588).

Item	Response range	Mean	SD	Median	IQR
Turnover Intention Scale	4–20	11.45	2.70	11	10, 13
I would prefer another more ideal job than the one I now work in	1–5	3.29	1.34	4	2, 4
I have thought seriously about changing hospitals since I began working here	1–5	2.42	1.18	2	1, 3
I hope to be working for this hospital until I retire (reverse scoring)	1–5	3.63	1.09	4	3, 4
I seriously intend to look for another job within the next year	1–5	2.10	1.10	2	1, 3

Abbreviations: IQR, interquartile ranges; SD, standard deviation.

### 3.4. Influencing Factors of the Turnover Intention of Participants

The final regression model explained 24.4% of the variance in turnover intention (*R* ^2^ = 0.244, Adjusted *R* ^2^ = 0.234, *F* = 24.051, *p* < 0.001), indicating an acceptable model fit. Variables with statistically significant differences in turnover intention were entered into the multiple linear regression model (Table 5). The regression results showed that married participants had lower turnover intention compared to single participants (*B* = −0.608, *p* = 0.008, 95% CI = −1.058, −0.159). Health status was significantly associated with turnover intention, with participants in fair (*B* = 0.507, *p* = 0.007) and poor health (*B* = 1.424, *p* < 0.001) having higher turnover intention compared to those with excellent health. Participants with good sleep quality showed lower turnover intention (*B* = −0.813, *p* < 0.001, 95% CI = −1.085, −0.542). Compared with participants with ≥ 15 years of experience as a midwife, those with less experience had higher turnover intention: < 5 years (*B* = 0.698, *p* = 0.002), 5–10 years (*B* = 0.503, *p* = 0.010), and 10–15 years (*B* = 0.789, *p* < 0.001). Turnover intention did not significantly differ across night shift modes (*p* > 0.05). Participants who needed to complete additional work during post‐night‐shift rest periods (*B* = 0.275, *p* = 0.039) and those who experienced job burnout (*B* = 0.714, *p* < 0.001) had higher turnover intention. Participants who were satisfied with the current rotation schedule (*B* = −1.342, *p* < 0.001) and their overall work (*B* = −0.356, *p* = 0.008) had lower turnover intention.

**TABLE 5:** Multiple linear regression analysis of factors influencing the turnover intention of midwives.

Variables	*B*	SE	95% CI	*β*	*t*	*p* value
Married	−0.608	0.229	−1.058, −0.159	−0.094	−2.655	0.008
Have one child and above	−0.066	0.209	−0.477, 0.344	−0.012	−0.318	0.751
Health status (Ref: Excellent)						
Good	0.034	0.186	−0.330, 0.398	0.006	0.184	0.854
Fair	0.507	0.188	0.139, 0.875	0.094	2.703	0.007
Poor	1.424	0.325	0.786, 2.062	0.113	4.376	< 0.001
Good sleep quality	−0.813	0.138	−1.085, −0.542	−0.141	−5.872	< 0.001
Hospital level (Ref: Public tertiary A hospital)						
Public tertiary B hospital	0.254	0.183	−0.106, 0.614	0.032	1.383	0.167
Public secondary hospital and below	0.109	0.162	−0.209, 0.427	0.015	0.672	0.502
Years of experience as a midwife (Ref: ≥ 15)						
< 5	0.698	0.227	0.251, 1.144	0.111	3.066	0.002
5∼	0.503	0.194	0.121, 0.884	0.090	2.585	0.010
10∼	0.789	0.201	0.395, 1.182	0.126	3.932	< 0.001
Night shift mode (Ref: Mode 1)						
Mode 2	0.210	0.141	−0.068, 0.487	0.039	1.482	0.138
Mode 3	0.202	0.217	−0.223, 0.628	0.024	0.932	0.351
Mode 4	0.469	0.283	−0.085, 1.024	0.040	1.660	0.097
Other tasks during the night shift	0.177	0.129	−0.077, 0.430	0.031	1.365	0.172
Finish night shift on time	−0.166	0.127	−0.416, 0.084	−0.031	−1.301	0.194
On‐call during post‐shift rest	0.180	0.130	−0.075, 0.436	0.033	1.384	0.167
Additional work during post‐shift rest	0.275	0.133	0.014, 0.536	0.051	2.068	0.039
Satisfied with rotation schedule	−1.342	0.137	−1.611, −1.073	−0.249	−9.790	< 0.001
Job burnout	0.714	0.167	0.386, 1.042	0.106	4.265	< 0.001
Overall job satisfaction	−0.356	0.135	−0.621, −0.091	−0.065	−2.636	0.008

*Note: B*: unstandardized coefficient *B*, 95% CI: 95% confidence interval of unstandardized coefficient *B*, *β*: standardized coefficient beta.

Abbreviation: SE, standard error.

## 4. Discussion

### 4.1. Turnover Intention Among Night‐Shift Midwives and International Comparison

Our findings revealed a turnover intention rate of 49.7% among night‐shift midwives in China. Because few studies use identical instruments to assess midwives’ turnover intention, direct comparison of scale scores is difficult. Therefore, the turnover intention rate can provide a general reference. The rate in this study was higher than in the Netherlands (33.3%) and Australia (42.8%) [30, 31], lower than in the United Kingdom (57%) [52], and significantly lower than in Ghana (87.2%) [33]. These comparisons should be interpreted cautiously, given potential differences in study populations, measurement approaches, midwifery practice models, and socioeconomic contexts.

Differences in working conditions and practice contexts may partly explain this variation. The present study specifically focused on night‐shift midwives working in hospital‐based or community settings, who are exposed to higher workload intensity, irregular schedules, and more complex clinical environments [15], compared with broader midwifery samples. These factors have been consistently associated with increased work stress and turnover intention. In addition, cross‐national differences in the midwifery practice model and socioeconomic contexts may further contribute to variation in turnover intention. In some countries, midwives primarily work in community‐based or self‐employed models with greater professional autonomy and flexible work arrangements [30, 37], whereas in others, including China, midwives mainly work in hospital settings with rotational shift systems and stricter organizational management [36, 53, 54]. Dissatisfaction with hospital‐based organizational environments has been identified as an important contributor to midwives’ turnover intention [31]. Finally, cross‐country differences in income levels, employment security, and career development opportunities may further shape turnover intention, particularly in low‐ and middle‐income settings [33, 34].

### 4.2. Individual Characteristics Associated With Turnover Intention

Marital status was significantly associated with turnover intention. Married night‐shift midwives reported lower turnover intention than single midwives, consistent with a previous study in China [55], although that study did not specifically focus on midwives. Some studies have suggested that family responsibilities may increase turnover intention [12, 30, 31], which appears to contradict our findings. However, marital status does not necessarily indicate family burden, and cultural differences may explain this inconsistency. Married midwives may receive more social support from their partners [56], and in the Chinese cultural context, women often prioritize job stability [57]. Financial responsibilities related to childcare, elder care, or housing may be associated with lower turnover intention, consistent with the lower turnover intention among married night‐shift midwives. Physical health factors also showed important associations with turnover intention. Night‐shift midwives with better health status had lower turnover intentions, consistent with previous research [37, 58]. Health status showed a clear gradient effect, with participants in poor health having significantly higher turnover intention compared to those with excellent health. Midwives with better health status may cope more effectively with the physical and mental stress of night shifts, whereas those with poorer health may experience reduced performance and subsequent rest, which may be associated with job satisfaction and turnover intention. Our results also found that individuals with better sleep quality had lower turnover intention, consistent with findings from studies among healthcare workers [59, 60]. For night‐shift midwives, good sleep quality may be important for restoring physical and mental fatigue caused by night shifts. Studies have shown that sleep quality is a crucial foundation for sustaining high‐intensity night shift work, and is also associated with job performance and clinical decision‐making [23, 61]. Poor sleep quality is associated with work mistakes and work burnout, which may also be related to turnover intention. Previous studies suggest that managers should consider midwives’ health status when designing rotation schedules, as poor health may be linked to sleep disorders and reduced recovery [62, 63].

### 4.3. Work‐Related and Organizational Factors Associated With Turnover Intention

The results showed that midwives with less than 15 years of experience had significantly higher turnover intention compared to those with ≥ 15 years of experience. This may be related to several factors. One possible explanation is that less experienced midwives tend to work more night shifts, as in many Chinese hospitals, midwives can reduce or stop night shifts after gaining more experience or higher positions. Although we did not analyze night shift frequency differences across experience groups in detail, frequent night shifts have been identified as an important factor associated with higher turnover intention [64]. Midwives in the early‐ and mid‐career stages may have fewer coping resources and find it more difficult to manage night‐shift stress. They are also more likely to perceive a poor work environment and experience job burnout, which has been associated with higher turnover intention [65]. Although night shift mode was not significantly associated with turnover intention in our regression analysis, the average night shift duration exceeds 12 h. In addition, the average rest period after some night‐shift modes is less than 2 days. Existing research indicates that at least two nights of rest are required after night shifts to recover effectively [66], suggesting that current arrangements may compromise recovery, though the direct link to turnover intention requires further investigation. Midwives who had to complete additional work during post‐night‐shift rest periods reported higher turnover intentions. These tasks included teaching or research that could not be completed during clinical hours, which essentially amounts to overtime. Overtime has been associated with higher turnover intention [67, 68] and reduced recovery and job satisfaction. Night‐shift midwives satisfied with their current rotation schedules had lower turnover intention, consistent with previous studies [12, 37]. Satisfied midwives may perceive night shift mode, interval time, and rest time as reasonable, whereas dissatisfied midwives may experience work scheduling challenges that increase turnover intention. Notably, job burnout prevalence among participants was high (79.8%), and those experiencing burnout had significantly higher turnover intention. Midwives dissatisfied with their overall work also reported higher turnover intention, consistent with the findings of other studies [31, 69]. These factors appear to be interconnected, as our findings suggest associations between work scheduling dissatisfaction, burnout, and overall job satisfaction. Ran et al. indicated that job burnout is positively associated with turnover intention and found relationships between job burnout and job satisfaction [70], which is consistent with our observations. The high burnout rate highlights the demanding nature of night shift midwifery work and suggests that addressing burnout may be a priority for retention strategies.

### 4.4. Implications and Recommendations

Considering the factors associated with turnover intention, our findings suggest that multi‐level interventions for night‐shift midwives may be beneficial. At the individual level, managers could consider optimizing shift scheduling based on departmental human resources and the personal circumstances of night shift midwives, taking into account individual characteristics such as marital status, health status, and sleep quality. It may be useful to explore night shift models, interval times between shifts, and rest periods suitable for both the department and midwives, including ensuring adequate recovery time (at least two consecutive days off after intensive night shifts) and limiting consecutive night shifts. Appropriate roles could be assigned, with efforts made to minimize overtime or provide fair compensation for extra work. At the organizational level, regular assessments of midwives’ scheduling satisfaction and overall job satisfaction could be conducted. Managers could also provide targeted training in health management, sleep management, and stress management to alleviate job burnout, which was highly prevalent in our sample. Particular attention may be needed for less experienced midwives and those required to complete additional non‐clinical work, ensuring they have sufficient rest time and support. At the system or policy level, it is possible that individual‐ or organizational‐level interventions alone may be insufficient if underlying structural factors are not addressed. While this study did not examine organizational factors such as staffing levels and workload distribution, these upstream determinants may significantly influence midwives’ health, job satisfaction, and turnover intention. Future research and policy initiatives could focus on ensuring adequate staffing ratios, equitable workload distribution, and supportive work environments that may help address factors associated with turnover intention.

## 5. Limitations

This study has several limitations that should be acknowledged. First, while convenience sampling was used across different hospital levels, the final sample was predominantly from public tertiary hospitals (69%) with highly educated participants (81.4% undergraduate degrees), which may limit the generalizability to midwives working in different healthcare settings or with varied educational backgrounds. In addition, because the survey link was disseminated through hospital managers and the total number of eligible midwives approached could not be determined, a response rate could not be calculated; therefore, selection bias and potential non‐response bias may have occurred. Second, the cross‐sectional design prevents the establishment of causal relationships between variables, requiring future longitudinal studies to determine temporal relationships and causal mechanisms. Third, several potentially relevant factors were not measured, including income levels, social and family support systems, psychological health indicators beyond burnout, workload intensity, and organizational culture factors, which may represent unmeasured confounders and bias the estimated associations. Fourth, self‐reported data may introduce recall bias and social desirability bias. Moreover, the classification of turnover intention using the sample mean as a cutoff is a methodological limitation, as it provides descriptive information but lacks theoretical and empirical validation for predicting actual turnover behavior and may lead to misclassification. Finally, this study did not examine potential mediating mechanisms between variables, and the findings may have limited generalizability to healthcare settings in other cultural contexts with different systems and values.

## 6. Conclusions

Nearly half of night‐shift midwives in mainland China reported turnover intention (49.7%). Being married, having better health status, good sleep quality, and higher satisfaction with shift scheduling and overall work were associated with lower turnover intention, whereas completing additional work during post‐shift rest time and job burnout were associated with higher turnover intention. These findings suggest that hospital administrators may consider optimizing shift scheduling, protecting rest time, and routinely monitoring burnout and job satisfaction to support retention and workforce sustainability.

## Funding

The authors received no specific funding for this work.

## Conflicts of Interest

The authors declare no conflicts of interest.

## Data Availability

The data that support the findings of this study are available from the corresponding author upon reasonable request.
